# Involvement of Sirtuins and Klotho in Cardioprotective Effects of Exercise Training Against Waterpipe Tobacco Smoking-Induced Heart Dysfunction

**DOI:** 10.3389/fphys.2021.680005

**Published:** 2021-07-20

**Authors:** Samaneh Sadat Alavi, Siyavash Joukar, Farzaneh Rostamzadeh, Hamid Najafipour, Fatemeh Darvishzadeh-mahani, Abbas Mortezaeizade

**Affiliations:** ^1^Neuroscience Research Center, Institute of Neuropharmacology and Cardiovascular Research Center, Institute of Basic and Clinical Physiology Sciences and Department of Physiology and Pharmacology, Afzalipour School of Medicine, Kerman University of Medical Sciences, Kerman, Iran; ^2^Cardiovascular Research Center, Institute of Basic and Clinical Physiology Sciences and Department of Physiology and Pharmacology, Afzalipour School of Medicine, Kerman University of Medical Sciences, Kerman, Iran; ^3^Physiology Research Center, Institute of Basic and Clinical Physiology Sciences, Kerman University of Medical Sciences, Kerman, Iran; ^4^Pathology and Stem Cell Research Center, Kerman University of Medical Sciences, Kerman, Iran

**Keywords:** waterpipe tobacco smoking, exercise training, cardiac function, apoptosis, sirtuins, Klotho

## Abstract

Despite its negative effect on the cardiovascular system, waterpipe smoking (WPS) is currently popular worldwide, especially among youth. This study investigated the effects of moderate endurance exercise on heart function of rats exposed to WPS and its possible mechanism. The animals were randomly divided into four groups: control group (CTL), the exercise group (Ex) which trained for 8 weeks, the waterpipe tobacco smoking group (S) exposed to smoke inhalation (30 min per day, 5 days each week, for 8 weeks), and the group that did exercise training and received waterpipe tobacco smoke inhalation together (Ex + S). One day after the last session of Ex and WPS, cardiac pressures and functional indices were recorded and calculated. The levels of SIRT1, SIRT3, Klotho, Bax, and Bcl-2 in the serum and heart, the expression of phosphorylated GSK3β of heart tissue, and cardiac histopathological changes were assessed. WPS reduced systolic pressure, +dP/dt max, -dP/dt max, and heart contractility indices (*P* < 0.001 vs. CTL) and increased cardiac tissue lesions (*P* < 0.05 vs. CTL) and end diastolic pressure and Tau index (*P* < 0.001 vs. CTL) of the left ventricle. Exercise training normalized the left ventricular end diastolic pressure, +dP/dt max, and contractility index. Also, exercise improved the levels of SIRT1, SIRT3, Klotho, and Bcl-2 and reduced Bax level in the heart. The findings showed that WPS causes left ventricular dysfunction. Moderate exercise prevented WPS-induced heart dysfunction partly through its anti-apoptotic features and activation of the sirtuins and Klotho pathways.

## Introduction

Tobacco, a product derived from the yellow and brown leaves of *Nicotiana tobacum*, is a plant widely cultivated in many countries around the world (Miri-Moghaddam et al., 2014). According to WHO, about 2.4 billion people worldwide smoke tobacco, including cigarettes, pipes, and hookah. It is also estimated that about 6.4 million deaths in 2015 were related to tobacco use, which could reach 8.3 million deaths by 2030 (Mathers and Loncar, 2006; Danaei et al., 2017; Nakhaee et al., 2020). The prevalence of hookah smoking in the Middle East is about 6–34% and in Iran ranges from 10.2 to 11.3% (21.4% in men and 1.4% in women) of the population, especially among adolescents (Ghasemian et al., 2015; Jawad et al., 2018). Human and animal studies indicated a significant association between hookah smoking and the prevalence of heart diseases (El-Zaatari et al., 2015; Qasim et al., 2019). An experimental study demonstrated that 8 weeks of waterpipe tobacco smoking is associated with the left ventricular systolic and diastolic dysfunction in rats (Nakhaee et al., 2019). Possible cardiac damage due to hookah smoking is attributed to inflammation, oxidative stress, and other factors that lead to disruption of energy metabolism, apoptosis, and change in gap junctions (Minicucci et al., 2016).

Exercise training is accepted as a non-pharmacological strategy to prevent cardiovascular diseases. Previous study demonstrated that swimming exercise training can attenuate the negative effect of waterpipe smoking on histopathological heart change and mechanical function. This effect partly applies through reduction of pro-inflammatory and increasing antioxidant factors (Nakhaee et al., 2019), however, the underlying molecular pathways need further investigation.

Sirtuins are a family of enzymes with seven paralogs (SIRT1-7) in mammals that catalyze NAD-dependent acetylation and/or ADP-ribosylation in proteins and they are known as oxidative stress sensors and cellular redox modulators (Michan and Sinclair, 2007; Matsushima and Sadoshima, 2015). Evidence suggests that SIRT1 and SIRT3 protect the heart against oxidative stress and ischemic heart disease (Ianni et al., 2018). On the other hand, it has been shown that cigarette smoke can reduce the level and activity of SIRT1 using oxidant-mediated mechanisms and potentiates the release of NF-κB-dependent pro-inflammatory cytokines in macrophages *in vitro* and *in vivo* (Yang et al., 2007).

Klotho is an anti-aging protein that extends life span through participation in many pathways such as regulation of cell-insulin sensitivity, Wnt (Wingless/int) signaling, calcium, and of phosphate homeostasis (Mencke et al., 2017). Research confirms that the Klotho protein effectively reduces heart cell damage and apoptosis, and is considered a potentially valuable cardioprotective agent (Olejnik et al., 2020). The results of a recent study show that SIRT1 cooperates with Klotho in improving pathological and physiological conditions (Najafipour et al., 2020). On the other hand, Klotho deficiency downregulates the expression of vascular SIRT1 and thus decreases its activity, and reciprocally reduction in SIRT1 activity mediates disorders due to Klotho shortage (Gao et al., 2016). Interestingly, different human and animal studies have indicated the involvement of Klotho (Baghaiee et al., 2018; Tan et al., 2018) and sirtuins (Donniacuo et al., 2019; Najafipour et al., 2020) in the beneficial effects of exercise training.

Glycogen synthase kinase-3β (GSK-3β), a multi-functional kinase and as a downstream part of the PI3K/AKT/GSK-3β signaling pathway, is a major regulator of cell growth, metabolism, and survival (Guertin and Sabatini, 2007). GSK-3β phosphorylation disturbance also has an important role in cardiac pathophysiology and exercise training via regulation of GSK-3β phosphorylation to protect myocardia against ischemic stress (Naderi-Boldaji et al., 2019).

Considering the mechanisms of deleterious effects of WPS on the heart are not clearly known, in present study, we investigated the effect of hookah consumption along with treadmill moderate training exercise on cardiac performance and also examined the hypothesis that the effect of exercise training may be partly mediated through SIRT1, SIRT3, Klotho, and phosphorylation of GSK-3β at Ser9.

## Materials and Methods

### Animals and Grouping

The experiment was conducted based on the national care and use of laboratory animals’ guidelines (ethics committee permission of Kerman University of Medical Sciences, Kerman, Iran; No*: IR.KMU.REC.1397.541.*). Chemical materials were purchased: sodium thiopental from Sandoz, Austria; SITR1, SIRT3, Bax, Bcl-2, and α-Klotho assay kits from Hangzhou Eastbiofarm, China; a cotinine ELISA kit from Sigma-Aldrich, United States; and a cardiac troponin I ELISA kit from Abcam, United States. Primary antibodies against Phospho-GSK-3β (Ser9) and its secondary antibody were obtained from Cell Signaling Technology, United States, and PVDF membrane and enhanced chemiluminescence (ECL) detection kits were purchased from Roche, Germany. The Double Apple tobacco was the product of the *Al Fakher* Company of United Arab Emirates.

Fifty-two male Wistar rats weighing 180–220 g were kept in a room with controlled temperature (22–23°C), a 12 h dark and 12 h light cycle, and they had free access to water and standard chow. Then, they were divided into four groups of 13 including a sham-control group (CTL) exposed to room air during the study period, an exercise group (Ex) which trained for 8 weeks, a waterpipe tobacco smoking group (S) exposed to waterpipe tobacco smoke (30 min per day, 5 days each week, for 8 weeks), and a group that underwent exercise training and inhaled waterpipe tobacco smoke together (Ex + S). Seven animals of each group were used for measurement of heart performance and blood pressure and the other six animals were used for biochemical and histopathological assessments.

### Moderate Intensity Exercise Training Protocol

Animals were trained on treadmills at 0° slope, 5 days/week for 8 weeks. The intensity and duration of exercise gradually increased. The speed and duration of running on the treadmill in the first week was 8 m per min for 10 min, in the second week was 12 m per min for 25 min, in the third week was 18 m per min for 40 min, and in the fourth to eighth week was 24 m per min for 60 min (Iwamoto et al., 1999). There was a manual shock network that was used rarely when needed.

### Waterpipe Smoking Protocol

In the WPS subgroups, 2 h after daily exercise training session, animals were exposed to waterpipe smoke, 5 days/week for 8 weeks. The smoking device used in this study was designed in our research laboratory and its details were explained previously (Nakhaee et al., 2019). Briefly, to simulate the conditions of WPS, a 50 × 30 × 15 glass enclosure for rats was used to inhale 10 g of hookah smoke for 30 min. Smoke and fresh air were introduced intermittently into the chamber as 30 s of smoke and 30 s of fresh air to clear out the smoke, and after an additional 30 s of breathing fresh air the next cycle would start. Cycles were repeated 20 times (20 × 1.5-min exposure = 30-min exposure session) daily. Animals in the CTL group were housed in the chamber for an equal amount of time as the test groups to simulate the stress of the protocol environment. At all sessions of smoke inhalation, the level of carbon monoxide (CO) concentration was maintained at the same level [mean ± standard deviation (SD): 916 ± 122 parts per million (ppm)] and recorded by the Testo 310 (Germany) CO measurement apparatus (Nakhaee et al., 2019, 2020). This is an accepted laboratory model for smoking studies and mimics the clinical effects of smoking well (Al-Awaida et al., 2014; Miri-Moghaddam et al., 2014).

### Measurement of Ventricular Pressure and Cardiac Function Indices

Twenty-four hours after the last session of the protocol, each animal was weighed and then anesthetized with thiopental sodium (50 mg/kg). In order to measure ventricular pressure and heart function indices, a polyethylene cannula, filled with heparin saline, was inserted into the right carotid artery and guided into the left ventricle. The catheter was connected to the pressure transducer and ventricular pressures were measured, after a stabilization period. The trachea of the animal was cannulated in order to attach an artificial ventilator if it was needed.

The left ventricular systolic pressure (LVSP), end diastolic pressure (LVEDP), maximal positive changes in left ventricular pressure (+dP/dt max, a contractility velocity index), maximum rate of reduction in left ventricular pressure (-dP/dt max, a relaxation velocity index), contractility index [+max dP/dt divided by pressure (P) at the time of maximum change with the dimension of 1/s], and Tau (left ventricular relaxation time constant as a relaxation index of the heart) indices were measured and calculated (Nakhaee et al., 2019).

### Tissue and Serum Preparation for Biochemical and Histological Examination

Twenty-four hours after the last session of the protocol, blood samples were taken and placed to clot for 15 min at 4°C; then serum samples of blood were separated using a centrifuge with an intensity of 3,500 rpm for 15 min and stored at −20°c for biochemical measurement of serum cardiac troponin I (cTnI), as an index of cardiac injury, SIRT1 and SIRT3 enzymes, Bax, and Bcl-2 as apoptosis-related factors. The apex of the animal’s heart was taken and immediately frozen in liquid nitrogen and stored at −80°C for assessment of SIRT1, SIRT3, Bax, Bcl-2, Klotho, and *p*-GSK-3β protein levels. The rest of the heart was used for histological examination.

### Histological Examination of the Heart

The rest of the heart was fixed in 10% formalin buffer. Then, it was placed in paraffin after dehydration by alcohol (gradually 80–100%) and xylene. The 5-μm slides were prepared from paraffin blocks and stained with hematoxylin and eosin. Slides were investigated by a pathologist who was blind to the animal group. Histopathological changes were classified into five degrees, including unchanged or negative (−), minimal (+) (local myocyte lesions), mild (++) (multifocal destruction with mild degrees of inflammatory processes), moderate (+++) (widespread degradation of myofibrils or diffuse inflammatory processes), and severe (++++) (marked) (necrosis + diffuse inflammatory processes) (Karthikeyan et al., 2007).

### Western Blot Analysis

Western blotting was used to measure GSK-3β protein in phosphorylated forms. Twenty-four hours after the last day of the protocol, a part of the heart apex was collected and stored at −80°C. After collecting all the samples, homogenization was performed in the protein extraction buffer. Next, the homogenized tissue was centrifuged at 4°C at 13,000 rpm for 15 min. Total protein concentration in heart samples were determined by the Bradford method. A total of 30 mg of protein from each sample was electrophoresed on 12.5% SDS-PAGE gel. After electrophoresis, the separated proteins in the gel were transferred to PVDF paper. The membranes were then incubated with the primary antibody for *p*-GSK-3β protein. Primary antibody binding was identified by the secondary antibody. Finally, they were identified using the ECL-2 substrate, and Gel-Dock (Bio-Rad company, United States) was used to photograph the membranes. The density of *p*-GSK-3β bands related to GAPDH were calculated. Image J software was used for analysis (Naderi-Boldaji et al., 2019).

### Enzyme−Linked Immunosorbent Assay

The ELISA method was used to measure cotinine and cardiac troponin I in serum, SIRT1, SIRT3, Bax, Bcl-2, and Klotho protein levels in tissue and serum (Lequin, 2005). For molecular measurement, a part of the apex was removed and washed with frozen cold phosphate buffer (PBS) (pH: 7.4). To evaluate SIRT1, SIRT3, Bax, Bcl-2, and Klotho, 50 mg of heart tissue was homogenized on ice and was centrifuged at 13,000 rpm for 15 min at 4°C. The supernatant was used for supplementary analysis. Total protein concentration was measured by the Bradford method. Quantitative value of each protein was measured using relevant ELISA kits based on the principle of competitive enzyme immunoassay and the biotin double antibody sandwich technology according to the manufacturer’s instructions.

### Statistical Analysis

Values are reported as mean ± standard error of the mean (SEM). Data analysis was performed by SPSS, version 20 (SPSS Inc., Chicago, IL, United States). The hypothesis of normality was assessed using the Shapiro-Wilk test. Statistical comparisons were performed by one-way ANOVA and Tukey post-hoc test. Differences of pathological scores were analyzed by Kruskal-Wallis test and Mann–Whitney test. The significance level was considered to be 5% (*p* < 0.05).

## Results

### Cotinine and Troponin I

**FIGURE 1:**
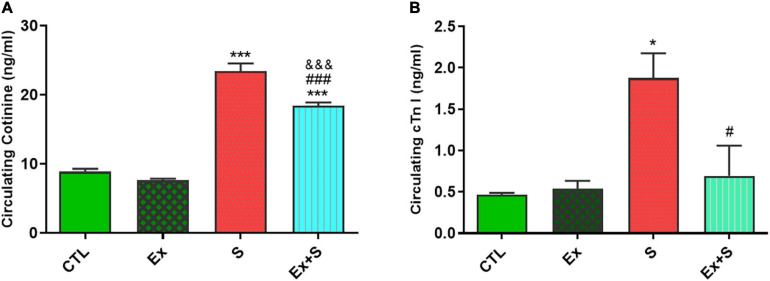
Effects of waterpipe smoke inhalation on serum cardiac troponin I **(A)** and cotinine levels **(B)** in experimental groups. CTL, control group; Ex, group subjected to exercise training for 8 weeks; S, group subjected to waterpipe tobacco smoke inhalation for 8 weeks; Ex + S, group subjected to exercise training and waterpipe tobacco smoke inhalation for 8 weeks. The results are presented as mean ± SEM, *n* = 7.*P < 0.05; ***P < 0.001 vs. CTL; ^#^*P* < 0.05, ^###^*P* < 0.001 vs. S; ^&&&^*P* < 0.001 vs. Ex.

Serum cotinine level, as an indicative marker for smoke exposure status, increased in groups who inhaled smoke (*P* < 0.001 vs. CTL). Although in the group that both trained and inhaled smoke, level of serum cotinine was lower (*P* < 0.001 vs. S group) but it was still higher in comparison with the control group (*P* < 0.001; Figure 1A).

Results show that WPS significantly increased cTnI level in the smoke inhalation group (*P* < 0.05 vs. CTL), and the coincidence of exercise along with smoke inhalation significantly decreased its level in comparison with the S group (*P* < 0.05). There was no significant difference between concentration of cTnI in the Ex and CTL groups (Figure 1B).

### Cardiac Pressures and Function

**FIGURE 2:**
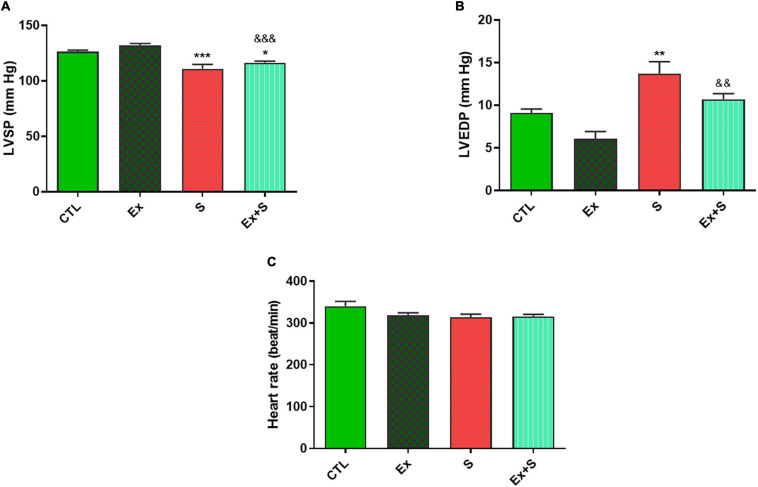
Effects of waterpipe smoke inhalation on left ventricular systolic pressure (LVSP) **(A)**, left ventricular end diastolic pressure (LVEDP) **(B)**, and heart rate **(C)** in experimental groups. CTL, control group; Ex, group subjected to exercise training for 8 weeks; S, group subjected to waterpipe tobacco smoke inhalation for 8 weeks; Ex + S, group subjected to exercise training and waterpipe tobacco smoke inhalation for 8 weeks. The results are presented as mean ± SEM, *n* = 7. *P < 0.05; **P < 0.01; ***P < 0.001 vs. CTL; ^&&^*P* < 0.01, ^&&&^P<0.001 vs. Ex.

The data of LVSP, LVEDP, and heart rate in all experimental groups are shown in Figure 2.

WPS decreased LVSP (*p* < 0.001) and increased LVEDP (*P* < 0.01) compared with the CTL group. On the other hand, the exercise group showed more stability in end diastolic pressure, with no significant difference between this parameter in the Ex and CTL groups (Figures 2A,B).

**FIGURE 3:**
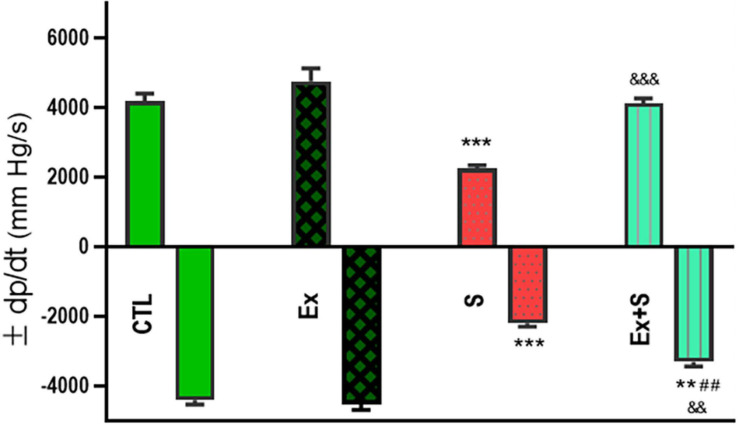
Effects of waterpipe smoke inhalation on positive dP/dt max (+dP/dt max) and negative dP/dt max (-dP/dt max) in experimental groups. CTL, control group; Ex, group subjected to exercise training for 8 weeks; S, group subjected to waterpipe tobacco smoke inhalation for 8 weeks; Ex + S, group subjected to exercise training and waterpipe tobacco smoke inhalation for 8 weeks. The results are presented as mean ± SEM, *n* = 7. **P < 0.01; ***P < 0.001 vs. CTL; ^##^*P* < 0.01 vs. S; ^&&^*P* < 0.01; ^&&&^*P* < 0.001 vs. Ex.

**FIGURE 4:**
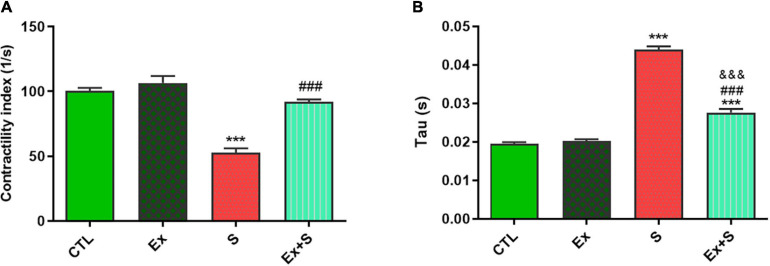
Effects of waterpipe smoke inhalation on contractility index **(A)** and Tau index **(B)** in experimental groups. CTL, control group; Ex, group subjected to exercise training for 8 weeks; S, group subjected to waterpipe tobacco smoke inhalation for 8 weeks; Ex + S, group subjected to exercise training and waterpipe tobacco smoke inhalation for 8 weeks. The results are presented as mean ± SEM, *n* = 7. ***P < 0.001 vs. CTL; ^###^*P* < 0.001 vs. S; ^&&&^*P* < 0.001 vs. Ex.

Smoke inhalation caused a significant reduction in values of +dP/dt max and -dP/dt max indices, contractility index, and Tau, in comparison with the CTL group (*P* < 0.001). Exercise along with smoke inhalation adjusted these parameters in the way that exercise-trained rats had markedly greater contractility, +dP/dt max, and -dP/dt max values (*p* < 0.01 vs. S). This positive effect was more prominent in cardiac contractility and +dP/dt max levels that were completely maintained in the CTL group (Figures 3, 4).

### Biochemical Parameters

**FIGURE 5:**
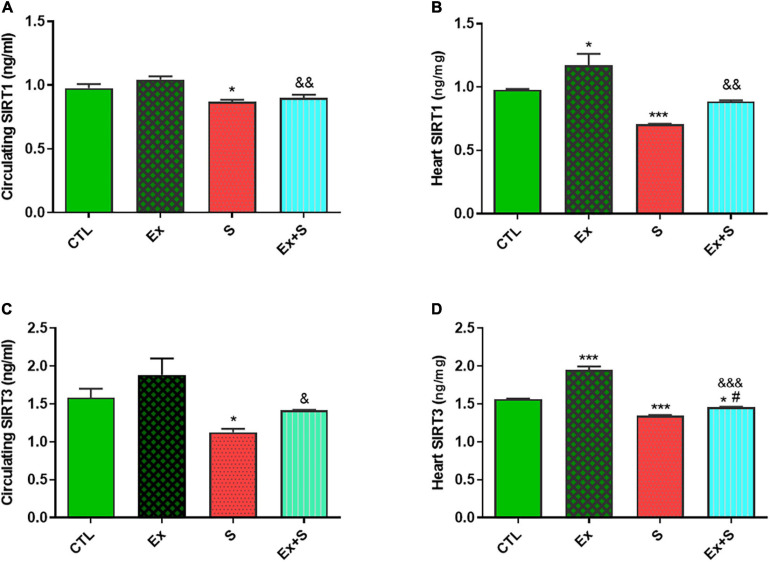
Effects of waterpipe smoke inhalation on sirtuin 1 (SIRT1) levels in serum **(A)** and in the heart **(B)**, and sirtuin 3 (SIRT3) levels in serum **(C)** and in the heart **(D)** in experimental groups. CTL, control group; Ex, group subjected to exercise training for 8 weeks; S, group subjected to waterpipe tobacco smoke inhalation for 8 weeks; Ex + S, group subjected to exercise training and waterpipe tobacco smoke inhalation for 8 weeks. The results are presented as mean ± SEM, *n* = (6–7). *P < 0.05; ***P < 0.001 vs. CTL; ^#^*P* < 0.05 vs. S; ^&^*P* < 0.05; ^&&^*P* < 0.01; ^&&&^*P* < 0.001 vs. Ex.

Data analysis clearly showed that 8 weeks of WPS significantly decreased both serum and heart tissue levels of SIRT1 in comparison with the CTL group (*P* < 0.05 and *P* < 0.001, respectively). Accompanying moderate intensity exercise training with smoke inhalation reversed this trend (*p* < 0.05, Ex + S vs. S group) so that there was no significant difference in SIRT1 between the Ex + S and CTL groups, however, the values were lower in the Ex + S group than the Ex group (*P* < 0.01; Figures 5A,B). Also, one-way ANOVA analyses showed that in the heart and serum of the S group, the level of SIRT3 was significantly lower than the CTL group (*P* < 0.5 and *P* < 0.001), and this was also normalized by exercise (Figures 5C,D).

**FIGURE 6:**
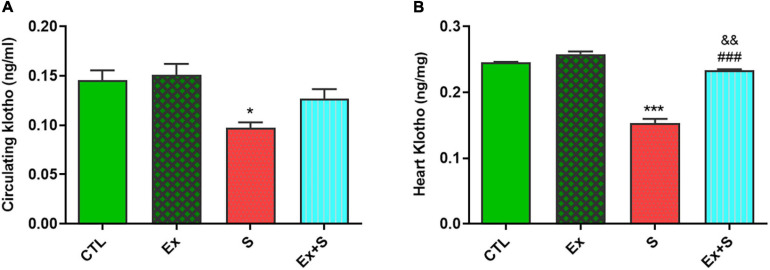
Effects of waterpipe smoke inhalation on α-Klotho levels in serum **(A)** and in the heart **(B)** in experimental groups. CTL, control group; Ex, group subjected to exercise training for 8 weeks; S, group subjected to waterpipe tobacco smoke inhalation for 8 weeks; Ex + S, group subjected to exercise training and waterpipe tobacco smoke inhalation for 8 weeks. The results are presented as mean ± SEM, *n* = (6–7). *P < 0.05; ***P < 0.001 vs. CTL; ^###^*P* < 0.001 vs. S; ^&&^*P* < 0.01 vs. Ex.

WPS significantly decreased the α-Klotho levels in serum (*p* < 0.05) and myocardium (*p* < 0.001 vs. CTL), while exercise intervention recovered this parameter to the level of the CTL group (Figure 6).

**FIGURE 7:**
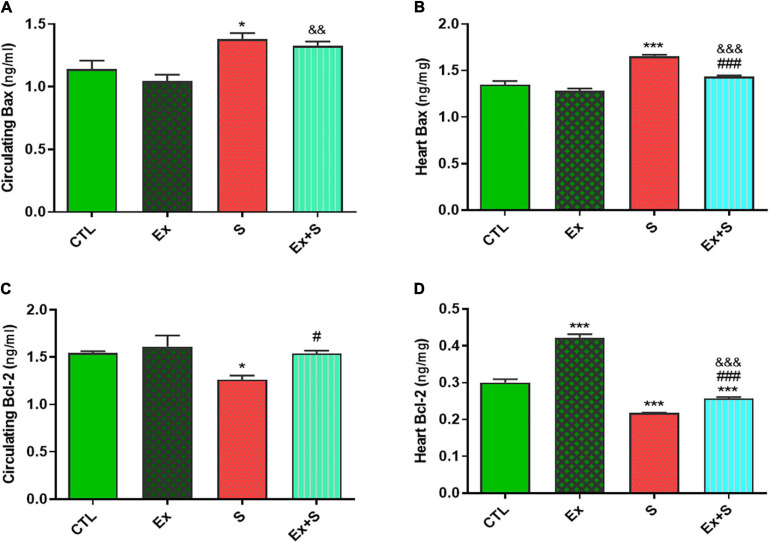
Effects of waterpipe smoke inhalation on Bax levels in serum **(A)** and in the heart **(B)**, and Bcl-2 levels in serum **(C)** and in the heart **(D)** in experimental groups. CTL, control group; Ex, group subjected to exercise training for 8 weeks; S, group subjected to waterpipe tobacco smoke inhalation for 8 weeks; Ex + S, group subjected to exercise training and waterpipe tobacco smoke inhalation for 8 weeks. The results are presented as mean ± SEM, *n* = (6–7). *P < 0.05, ***P < 0.001 vs. CTL; ^#^*P* < 0.05, ^###^*P* < 0.001 vs. S; ^&&^*P* < 0.01, ^&&&^*P* < 0.001 vs. Ex.

**FIGURE 8:**
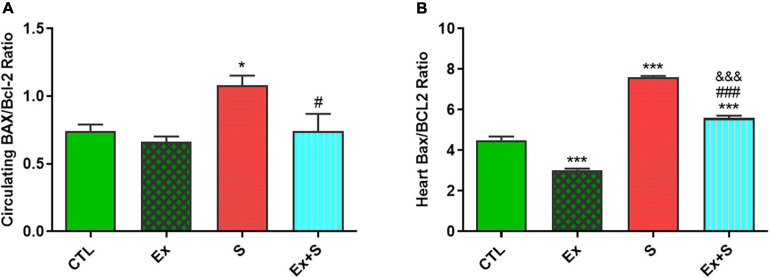
Effects of waterpipe smoke inhalation on Bax/Bcl-2 ratio in serum **(A)** and in the heart **(B)** in experimental groups. CTL, control group; Ex, group subjected to exercise training for 8 weeks; S, group subjected to waterpipe tobacco smoke inhalation for 8 weeks; Ex + S, group subjected to exercise training and waterpipe tobacco smoke inhalation for 8 weeks. The results are presented as mean ± SEM, *n* (6–7). *P < 0.05, ***P < 0.001 vs. CTL; ^#^*P* < 0.05, ^###^*P* < 0.001 vs. S; ^&&&^*P* < 0.001 vs. Ex.

WPS was also associated with an increase in the serum level and heart expression of Bax protein as an indicator of apoptosis (*P* < 0.05 and *P* < 0.001 vs. CTL group, respectively), while it decreased Bcl-2 expression in the heart (*P* < 0.001 vs. CTL) and serum (*p* < 0.05 vs. CTL). Exercise training along with WPS attenuated the effects of smoke inhalation on Bax and Bcl-2 values in both tissue and serum as it recovered the levels of Bcl-2 and decreased the levels of Bax (Figure 7). Also, results showed that WPS increased the Bax/Bcl-2 ratio and exercise training could normalize this ratio in serum and heart tissue (Figure 8).

### Expression of Glycogen Synthase Kinase 3β Phosphorylated at Ser9 (pS9-GSK-3β)

**FIGURE 9:**
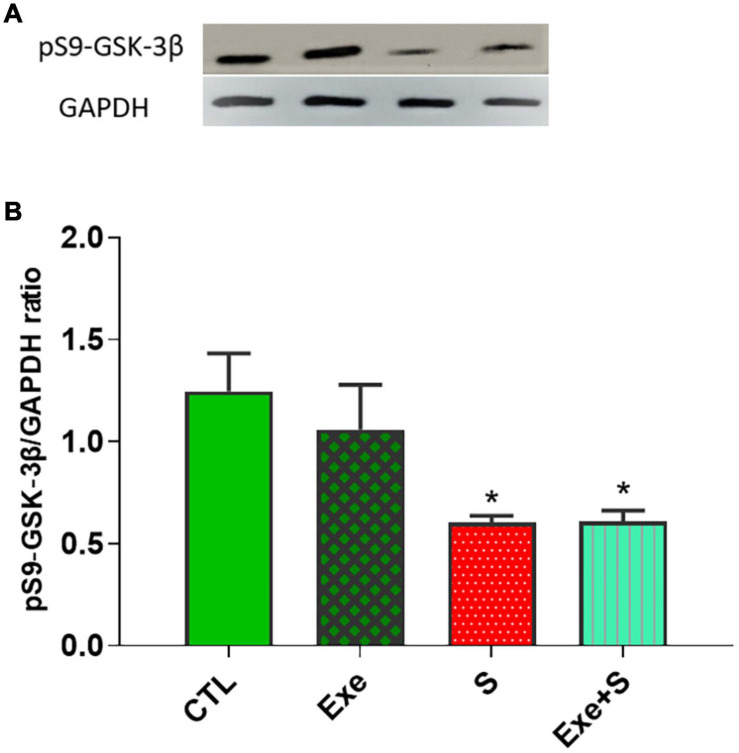
Effects of waterpipe smoke inhalation on the expression of cardiac pS9-GSK-3β proteins **(A)** and pS9-GSK-3β/ GAPDH ratio **(B)** in different experimental groups. CTL, control group; Ex, group subjected to exercise training for 8 weeks; S, group subjected to waterpipe tobacco smoke inhalation for 8 weeks; Ex + S, group subjected to exercise training and waterpipe tobacco smoke inahaltion for 8 weeks. The results are presented as mean ± SEM, *n* = (6–7). *P < 0.05 vs. CTL.

Figure 9 shows the expression of cardiac pS9-GSK-3β protein in experimental groups. WPS significantly decreased the expression of this protein in heart tissue (*p* < 0.05 vs. CTL group), although exercise could not recover from this negative effect of smoke inhalation.

### Histological Findings

**FIGURE 10:**
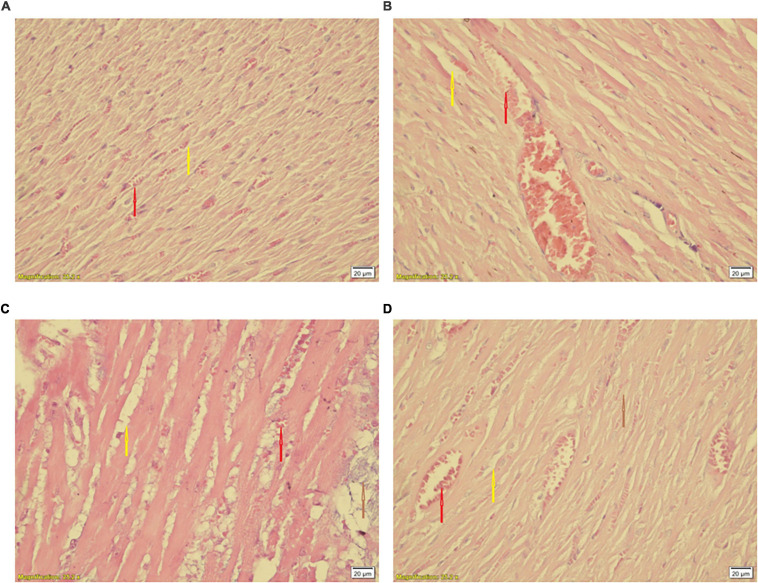
Histological changes of the heart in experimental groups. **(A)** CTL, **(B)** Ex, **(C)** S, and **(D)** Ex + S. Heart tissues were stained with hematoxylin and eosin and visualized under a light microscope. CTL, control group; Ex, group subjected to exercise training for 8 weeks; S, group subjected to waterpipe tobacco smoke inhalation for 8 weeks; Ex + S, group subjected to exercise training and waterpipe tobacco smoke inhalation for 8 weeks. Histopathological examination was performed under light microscopy at a magnification of ×40. Yellow arrow: interstitial edema, red arrow: congestion, brown arrow: cellular degeneration.

Statistical comparison of histopathological scores showed significant difference among hearts of the CTL and S groups (*P* < 0.05 vs. CTL) (Table 1). Smoke inhalation induced mild to moderate cardiac damage so about 40% of the animals’ hearts in the WPS-treated groups showed widespread degradation of myofibrils or diffuse inflammatory processes and about 30% showed multifocal destruction with mild degrees of inflammatory processes (Figure 10B) as compared to the normal group (Figure 10A). Despite some degree of reduction, moderate intensity exercise training did not prevent the negative histopathological alterations of cardiac muscle induced by waterpipe tobacco smoking (Table 1 and Figure 10).

**TABLE 1:** Histopathological scores and animal number with different degrees of injury in each group.

Groups	Myocardial	Pathology	Score			

	No change	+	++	+++	++++	Mean
CTL (*n* = 5)	4	1	0	0	0	0.16
Ex (*n* = 5)	4	1	0	0	0	0.16
S (*n* = 6)	1	1	2	3	0	2*
Ex + S (*n* = 6)	2	1	0	3	0	1.6*

n, number of animals; CTL, control group; Ex, group which was subjected to exercise training for 8 weeks; S, group which was subjected to waterpipe tobacco smoke inhalation for 8 weeks; Ex + S, group which was subjected to exercise training and waterpipe tobacco smoke inhalation for 8 weeks. (0) No change, (1) minimum (focal myocytes damage), (2) mild (small multifocal degeneration with slight degree of inflammatory process), (3) moderate (extensive myofibrillar degeneration and/or diffuse inflammatory process), and (4) sever (necrosis with diffuse inflammatory process), *P < 0.05 vs. CTL.

## Discussion

The aim of this study was to investigate the effects of moderate intensity exercise training on the heart of rats exposed to WPS and its possible mechanism. We showed that tobacco consumption causes pathological cardiac dysfunction by altering the levels of SIRT1, SIRT3, Klotho, *p*-GSK3β, and apoptotic pathways that affected the structure and function of the myocardium. Exercise attenuated the severity of heart injury, and systolic and diastolic dysfunction, and also maintained the left ventricular pressures at near the level of normal values. These effects were associated with a reduction in plasma troponin I and improvement of SIRT1, SIRT3, and α-Klotho levels in the heart tissue and serum. It also inhibited heart cell apoptosis by reducing the levels of Bax and increasing Bcl-2, respectively.

Cotinine is a tobacco alkaloid and is the major metabolic component of nicotine. An increased level of this marker is an index of exposure to tobacco smoke (Nakhaee et al., 2020), and in the present study, its increase in the S and Ex + S groups confirms the smoking method. However, this metabolite was significantly lower in the Ex + S group than the S group. A possible reason may be due to the link of stress hormones such as cortisol on nicotine metabolism (al’Absi et al., 2004). The modulatory effect of exercise on cortisol leads to the reduction of the cotinine level in the Ex + S group (Al-Eisa et al., 2016).

Previous studies have been shown that shisha tobacco and coal have negative effects on mechanical cardiac function (Khoramdad et al., 2020). Based on our previous findings, chronic WPS causes left ventricular dysfunction and cardiomyocytes injury, and combining WPS with swimming exercise prevents these negative effects (Nakhaee et al., 2019). In the present study, the decreased LVSP and increased LVEDP, and the reduction of positive and negative dP/dt max and contractility indexes, histopathological findings and an increase in the plasma level of cTnI, as an important biomarker of cardiac injury, also emphasized that passive smoking of waterpipe tobacco has deleterious effects on heart function. Moderate intensity exercise training clearly prevented the above-mentioned tobacco-induced disorders (Figures 2–4). The harmful effects of WPS could be attributed to its pathological impacts on ventricular tissue. Previous studies also reported that waterpipe smoke causes structural changes in the heart tissue that negatively affect the capacity of the heart muscle to pump blood, and may cause heart attacks due to the accumulation of free radicals and tissue inflammation (Al-Awaida et al., 2015; DiGiacomo et al., 2019). Exercise, probably through attenuation of oxidative stress and balance in the redox system, decreases heart injures (Nakhaee et al., 2019).

Our findings indicated that tobacco consumption reduces the proteins involved in cell survival including SIRT1, SIRT3, and Klotho, and increases the rate of apoptosis (Figures 5–8). Many cell functions, such as proliferation, apoptosis, cell survival, and response to stress, are mediated by class III histone deacetylases enzymes through deacetylating target proteins (Kiziltunc et al., 2019). A number of studies have reported that SIRT1, one of the members of class III histone deacetylases enzymes, inhibits apoptosis in cardiomyocytes (Tanno et al., 2010; Yu et al., 2016) and its reduction is associated with various cardiovascular pathologies such as cardiac hypertrophy and the severity of heart failure (Kong et al., 2010; Sosnowska et al., 2017). During stress, SIRT3, another member of this family, has similar effects. Results of Du et al. (2017) demonstrated that SIRT3 decreases the level of ROS and apoptosis in myocardial cells, and so has cardioprotective effects (Du et al., 2017). In agreement with other results (Nakhaee et al., 2019, 2020), our findings revealed that exercise improves cardiac dysfunction caused by smoking, probably by increasing the levels of SIRT1 and SIRT3. During exercise, increased ATP demand increases NAD+ levels and NAD+/NADH ratios, which provide more substrates for SIRT1 and SIRT3 (White and Schenk, 2012). It has been reported that both acute resistance exercise and endurance training induce anti-apoptotic and antioxidant activity by activating the SIRT1 (Suwa et al., 2008) and SIRT3 pathways, which reduce heart damage (Ferrara et al., 2008), and significantly improves the left ventricular contractile potency (Khakdan et al., 2020).

Klotho is a mediator that has ameliorative effects on inflammation, oxidative stress, and apoptosis. Similar to SIRT1 and SIRT3, the results of this study show that moderate intensity exercise training is sufficient to change the serum and myocardial level of Klotho. Therefore, its preventive effect probably mediates through modulation of the abovementioned process. It has been demonstrated that a period of exercise activates a variety of factors that increase the α-Klotho gene expression (Fritzen et al., 2016; Tanimura et al., 2016) and increases the serum level of S-Klotho in animal models and humans (Phelps et al., 2013). It should be noted that the intensity and duration of exercise are influential factors in Klotho expression (Najafipour et al., 2020).

Findings of the present study indicated the same direction variations in the levels of sirtuins and Klotho following tobacco and exercise, which suggest their relationship. The protective effects of exercise may be mediated by cooperation of both pathways. Other studies have also indicated the association between Klotho and SIRT1. It has been demonstrated that Klotho is an upstream regulator of SIRT1. Klotho’s deficiency reduces SIRT1 expression and activity in the arteries and therefore causes cardiovascular problems (Gao et al., 2016).

The Bcl-2 family, including the anti-apoptotic protein Bcl-2 and the proapoptotic protein Bax, can mediate apoptosis by modulating mitochondrial permeability and so are the central regulators of the intrinsic apoptotic cascade (Wilkins et al., 2012; Bai et al., 2020). Our findings showed that WPS activated apoptotic pathways and increased the Bax/Bcl-2 ratio, and exercise training could normalize the levels of Bax and Bcl-2 and their ratio in serum and heart tissue. In accordance with the results of the present study, it is reported that each tobacco smoking session results in about 1.04–7.75 mg of nicotine and there is evidence that nicotine can cause cardiomyocyte apoptosis (Bodas et al., 2016). On the other hand, endurance training reduces the apoptotic process in cardiomyocytes of an animal model (Kwak et al., 2006).

This study also revealed that tobacco smoking significantly decreased the expression of phosphorylated GSK3β as a cardioprotective factor in cardiac tissue (Figure 9).

Our previous study indicated that exercise training by increased GSK-3β phosphorylation protected the heart against isoproterenol-induced myocardial injury (Naderi-Boldaji et al., 2019). However, in the present study, exercise could not recover the reducing effect of tobacco smoking on phosphorylated GSK3β. The reason for this discrepancy needs further studies.

Pathological changes in the heart may be due to an imbalance of mediators involved in the apoptotic and anti-apoptotic pathways, leading to heart dysfunction. Our data showed that exercise was able to alleviated the Bax/Bcl-2 ratio in the hearts of animals exposed to WPS, therefore, exercise presumably can prevent heart dysfunction caused by WPS, which is consistent with the results of our previous studies (Naderi-Boldaji et al., 2019).

## Conclusion

The findings showed that tobacco smoking causes left ventricular dysfunction. We revealed the role of moderate intensity exercise training as a key factor in preventing smoke-induced cardiovascular injury and molecular mechanisms disturbance. Results of this study confirm that exercise-induced cardiac protection involves its anti-apoptotic features and activation of the sirtuins and Klotho pathways.

## Data Availability Statement

The raw data supporting the conclusions of this article will be made available by the authors, without undue reservation.

## Ethics Statement

The animal study was reviewed and approved by ethics committee permission of Kerman University of Medical Sciences, Kerman, Iran.

## Author Contributions

SJ devised the main conceptual ideas and designed the study. All authors were contributed in the acquisition, analysis or interpretation of data and drafting of the manuscript. All authors approved the final version of the manuscript.

## Conflict of Interest

The authors declare that the research was conducted in the absence of any commercial or financial relationships that could be construed as a potential conflict of interest.
